# Single-Walled Zeolitic Nanotube–Poly(oxazoline) Nanocomposites as Heterogeneous Catalysts for Acid–Base Cascade Reactions

**DOI:** 10.1021/acs.langmuir.5c01067

**Published:** 2025-05-16

**Authors:** Wenyang Zhao, Anthony Vallace, Younhwa Kim, Christopher W. Jones

**Affiliations:** School of Chemical & Biomolecular Engineering, 1372 Georgia Institute of Technology, 311 Ferst Drive, Atlanta, 30332 Georgia, United States

## Abstract

Zeolites with a unique, 1-dimensional form factor were recently discovered – zeolite nanotubes (ZNTs). Here we describe the synthesis and characterization of NaH-ZNT-poly­(oxazoline) composites targeting liquid-phase acid–base cascade catalysis. NaH-ZNT, a one-dimensional zeolite analogue with mesoporosity (3–4 nm) associated with nanotubes and inherent Brønsted acid sites associated with the microporous zeolite domains, is functionalized with poly­(oxazoline)-based triblock copolymers with varying molecular weights (3–17 kDa). The composites are characterized using N_2_ sorption, STEM, FTIR, and elemental analysis, confirming successful grafting and preservation of the zeolite nanotube structure. The composites’ catalytic performance is evaluated through separate acid and base reactions, followed by a combined cascade of a deacetalization–Knoevenagel condensation for the synthesis of chalcone compounds. High initial reaction rates are demonstrated, but modest overall cascade product formation rates are observed, attributed to interactions between Brønsted acid sites and base amine groups that occur in the polymer-grafted systems. Physical mixtures of NaH-ZNT-SH and poly­(oxazoline)­s, lacking covalent linkages between ZNT and the polymer, support this supposition. This work demonstrates the potential of NaH-ZNT-poly­(oxazoline) composites for liquid-phase cascade catalysis for synthesizing compounds of potential medicinal interest, highlighting the benefits of the grafting-to approach as well as the need for further optimization of the catalytic performance.

## Graphical abstract

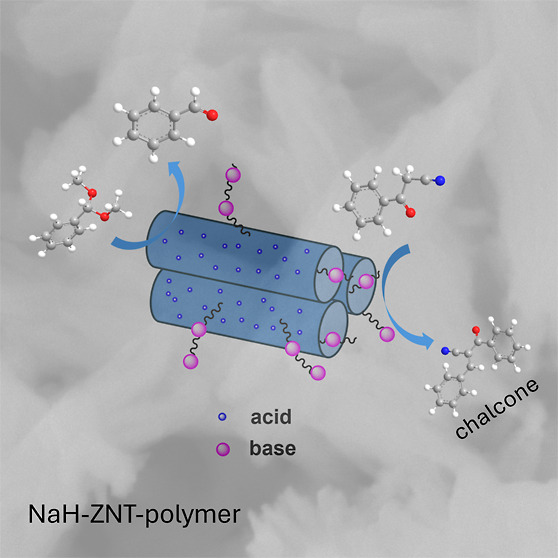

## Introduction

Cascade reactions are processes in which multiple chemical transformations occur consecutively within a single operational sequence or reaction setup, without the need to isolate intermediates. This efficient approach to chemical synthesis is highly sought after in various industries, including pharmaceuticals, petrochemicals, and materials science, due to its potential to streamline production, reduce waste, and lower costs.^1–3^ In the meantime, a variety of competing cascade reactions take place in living systems without interfering with each other, which is achieved through compartmentalization, where catalytic transformations and active sites, as well as reaction intermediates, are shielded within different compartments.^4,5^ Compartmentalized catalysts offer many advantages such as high reaction efficiency and atom economy, and it is possible to achieve multiple incompatible transformations within a single reaction medium.^6,7^

Over the past decades, various synthetic analogues have been designed to mimic living systems, with the major goal of maintaining spatial segregation of catalytic active sites. Similar to many enzyme complexes with antagonistic sites confined in separate domains, synthetic systems typically achieved site isolation via multiple-step, postsynthetic modifications to minimize the interaction between competing active sites. Multiple synthetic materials have been reported following this idea by active site grafting or functionalization, with a variety of platforms such as porous inorganic materials (e.g., silicas,^8–11^ zeolites,^12,13^ metal oxides,^14,15^ clays^16^), porous polymers,^17,18^ metal–organic frameworks,^19,20^ activated carbons,^21^ and so on. These materials typically have a high surface area with versatile pore structures and functionalities, easing modification efforts and providing abundant reaction surface(s) or sites during the catalysis. For example, Cleveland et al. combined both silica and polymeric domains to prepare silica–poly­(styrene) composites based on SBA-15 and MCM-41, and studied their activities for a two-step deacetalization–Knoevenagel reaction cascade. It was discovered that composites containing lower-molecular-weight polymers performed better due to the faster diffusion of the substrates.^8^ On the other hand, researchers have also developed polymer platforms including shell cross-linked micelles to compartmentalize incompatible active sites.^22–24^ In one study, Lee et al. synthesized amphiphilic poly­(2-oxazline) polymers as two-chamber nanoreactors for deacetalization–nitroaldol reactions. They demonstrated the confinement of the active sites within the micellar structure and achieved 99% substrate conversion with a yield of 86%.^25^

One potentially versatile approach to prepare compartmentalized catalysts involves zeolite–polymer composites. Zeolites are microporous aluminosilicates with high surface area, thermal stability, and unique pore structures, which can act as catalysts or catalyst supports.^26–28^ They also have well-defined pore sizes and shapes, making them ideal for facilitating complex reaction pathways. The Brønsted acidity of the proton-exchanged zeolites has been widely used for various heterogeneous catalytic reactions.^29–31^ However, the majority of natural and synthetic zeolites are microporous, 3-dimensional structures, limiting their potential for liquid-phase heterogeneous catalysis where bulky substrates are often involved. Decreasing the zeolite particle size can mitigate this issue by providing more external surface area. On the other hand, hierarchical zeolites containing mesopores are better alternatives, providing enhanced diffusivity and accessibility of active sites.^32^ Although several types of hierarchical zeolites, especially two-dimensional zeolites,^13,33,34^ have been explored for cascade catalysis, the applications of zeolites combined with polymers is relatively scarce. Recently, a hierarchical one-dimensional zeolite with a nanotubular structure was reported by Korde et al.^35^ These zeolite nanotubes consist of mesoporous channels with diameters of 3–4 nm and crystalline walls composed of a single layer. By integrating zeolite nanotubes with polymers via covalent bonding, additional functionalities can be incorporated to allow for the creation of tailored domains that can achieve cascade catalysis with high efficiency compared with their physical mixture counterparts.

In this work, multiple amine-modified poly­(oxazoline) triblock copolymers with molecular weights in the range of 3k to 17k Da were prepared and grafted onto proton-exchanged zeolite nanotubes (NaH-ZNT), and the composites were subsequently evaluated in the deacetalization–Knoevenagel condensation cascade test reaction. Different from the most commonly used Knoevenagel substrates such as malononitrile, ethyl cyanoacetate, or nitromethane, we chose to use benzoylacetonitrile because the cascade reaction can yield chalcone, which has been widely used as an effective template in medicinal chemistry for drug discovery.^36,37^ Additionally, it has been demonstrated that chalcone and its derivatives have a number of interesting biological properties such as antioxidant, cytotoxic, anticancer, antimicrobial, and so on.^38^ Hereby we demonstrated that using a grafting-to approach, the quenching between the acid sites of NaH-ZNT and the base sites of poly­(oxazoline)-NH_2_ can be alleviated to enhance the rate of the cascade reactions, and the composites have the potential to be used in pharmaceutical processes.

## Experimental Section

Chemicals used in this study, detailed synthetic methods, and characterization methods and tools are listed in the Supporting Information.

## Results and Discussion

### Synthesis and the Characterizations of the Single-Walled Zeolitic Nanotube-Polyoxazoline Nanocomposites

The detailed synthetic route for the preparation of NaH-ZNT-poly­(oxazoline) is illustrated in Scheme 1. Our objective was to explore the utility of ZNTs in the creation of multidomain catalysts applicable to liquid-phase, acid–base cascade catalysis and to study, in parallel, the interactions between the NaH-ZNT and poly­(oxazoline)­s. NaH-ZNT materials were synthesized following our previously reported literature.^35^ This newly discovered one-dimensional zeolite analogue has unique mesoporosity (3–4 nm) formed by a single layer of the beta and MFI building blocks, offering abundant Brønsted acidic sites (∼0.15 mmol/g) that can catalyze liquid-phase reactions.^39^ Moreover, the silanol groups (–OH) of the NaH-ZNT surface can be modified by a variety of silanes to offer versatile functional groups for polymer grafting. After NaH-ZNT synthesis, the surface was functionalized with thiol groups (–SH) by reacting with (3-mercaptopropyl) trimethoxysilane (MPTMS) (Scheme 1). The amount of surface –OH was estimated from the mass loss of dehydroxylation between 400 and 600 °C based on the TGA, and the amount of MPTMS used for the reaction was controlled so that it was stoichiometrically possible that all the Si–OH groups could react with silane to form Si–O–Si bonds, assuming an average reaction ratio of 2:1 (–OH/MPTMS).^40^ It is known that calcined NaH-ZNT slowly decomposes in aqueous media due to its ultrathin walls. Targeting complete conversion of surface –OH groups was done to increase the water stability of the NaH-ZNT, with the resulting material denoted as NaH-ZNT-SH. As shown in Figure 1a, the N_2_ sorption isotherm of NaH-ZNT appears to be type IV with a Type H3 hysteresis loop, indicative of the NaH-ZNT mesopore.^41^ The Type H3 loop is typically given by nonrigidly packed aggregates of particles,^41^ which is in close agreement with the NaH-ZNT particle morphology observed under STEM (Figures 2a and S1). The average pore size of NaH-ZNT was 3.8 nm derived from the DFT model, also in accordance with the previously reported value.^35^ After –SH modification, the BET surface area and DFT pore volume decreased because of the added mass from grafting. Moreover, the average pore size of NaH-ZNT-SH decreased to 3.5 nm (Table 1). Considering that the molecular size of MPTMS (0.6–0.7 nm)^42^ is much smaller than the NaH-ZNT mesopores, it is expected that the –SH modification happened both inside and outside of mesopores. Compared to the starting NaH-ZNT, the overall shape of the isotherms, as well as the particle morphology, did not change much after –SH modification, which indicates that the nanotube structure was retained (Figures 1a and 2a,b).

**Scheme 1:**
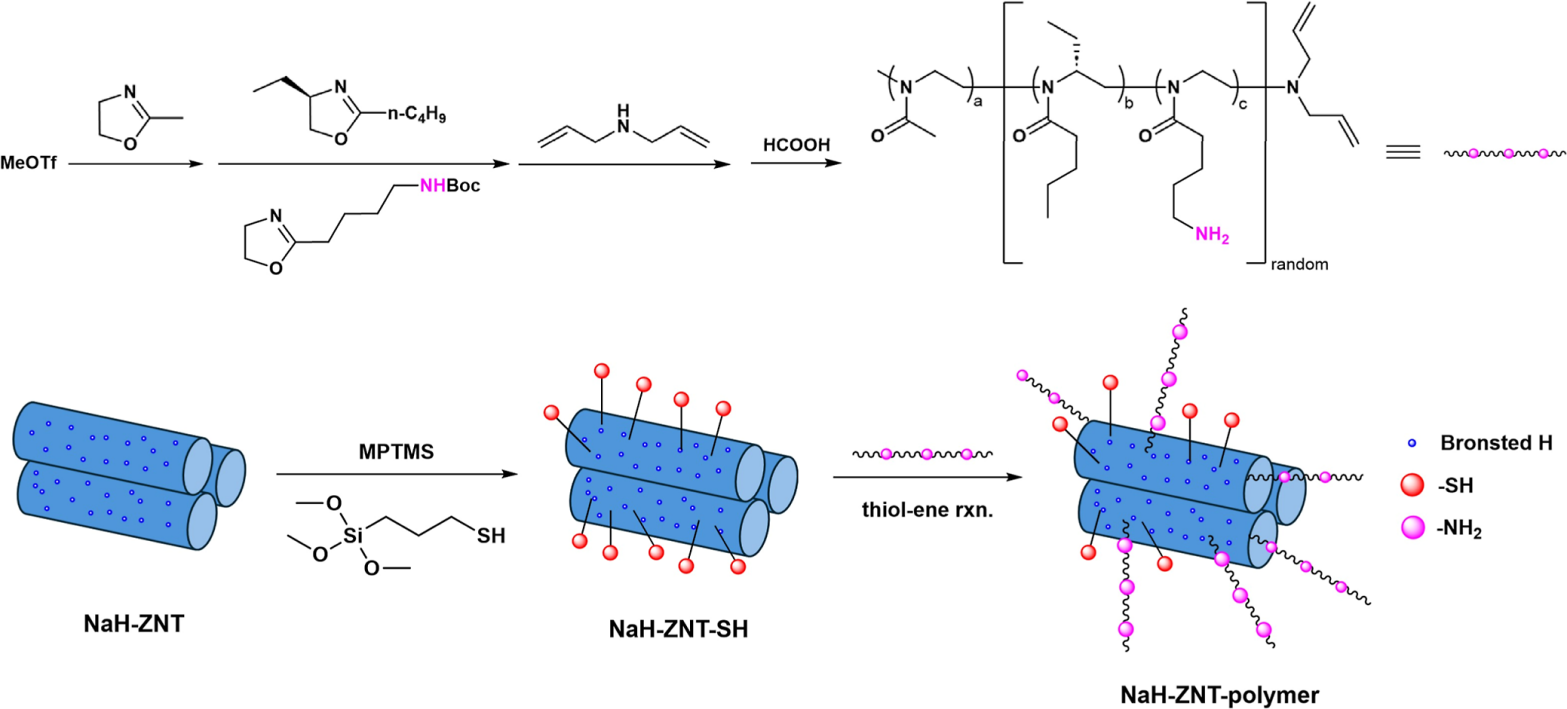
Illustration of the Synthetic Route of NaH-ZNT-Poly­(oxazoline) Composites^a^

a Cartoons do not represent the actual locations where polymers were grafted.

**Figure 1:**
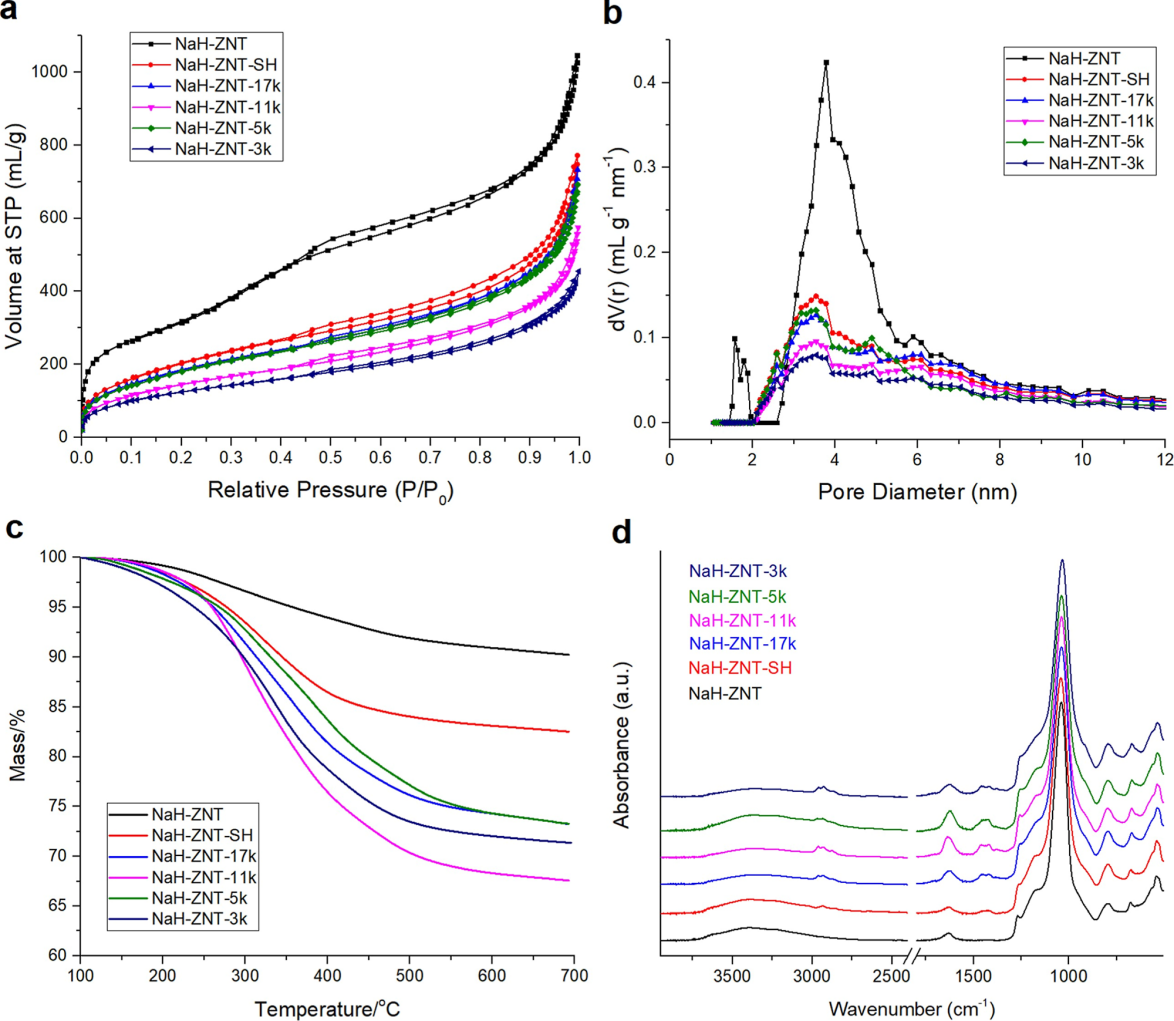
(a) N_2_ sorption isotherms, (b) DFT pore size distributions, (c) TGA curves, (d) FTIR spectra. Pore size distributions were calculated by the NLDFT method using a siliceous model.

**Figure 2:**
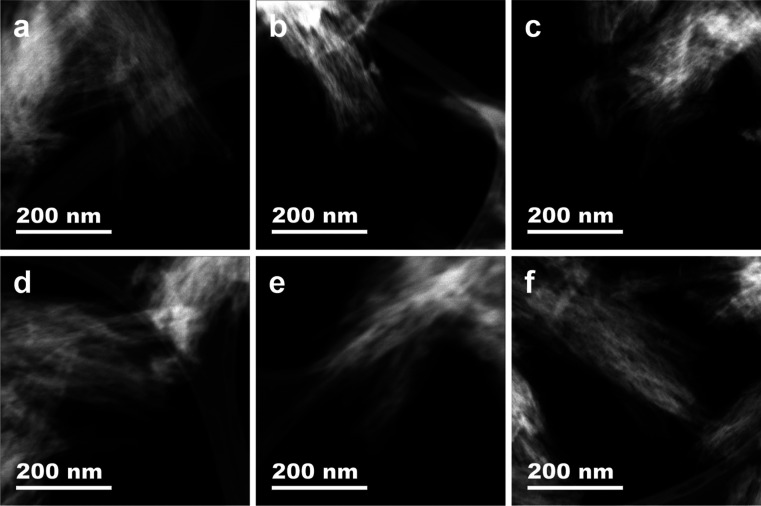
STEM images of (a) NaH-ZNT, (b) NaH-ZNT-SH, (c) NaH-ZNT-17k, (d) NaH-ZNT-11k, (e) NaH-ZNT-5k, (f) NaH-ZNT-3k.

**Table 1:** Textural Properties of the Samples^a^

sample	BET surface area (m^2^/g_Na‑ZNT_)	DFT pore volume (mL/g_Na‑ZNT_)	DFT average pore size (nm)
NaH-ZNT	1215	1.41	3.8
NaH-ZNT-SH	1025	1.35	3.5
NaH-ZNT-17k	961	1.31	3.5
NaH-ZNT-11k	840	1.15	3.5
NaH-ZNT-5k	945	1.19	3.5
NaH-ZNT-3k	650	0.86	3.5

a Pore volumes and average pore sizes were calculated by the NLDFT method using a siliceous model.

In the meantime, different oxazoline monomers were prepared following the reported procedure (Figure S2), and poly­(oxazoline)-based triblock copolymers were synthesized via cationic ring-opening polymerization (Scheme 1). The polymerization proceeded through nucleophilic propagation and was terminated via the addition of diallylamine, which generated ene-functionalized poly­(oxazoline)­s. These ene groups not only facilitate the determination of polymer molecular weight by performing end-group analysis by ^1^H NMR but, more importantly, act as anchoring sites when preparing the composite via the thiol–ene click reaction. Additionally, one of the three monomers carries a *tert*-butoxycarbonyl (Boc) protecting group, which after the polymerization, is cleaved to obtain free amine groups (–NH_2_) for basic catalysis. By treating the Boc-protected polymers in HCOOH, four poly­(oxazoline)-NH_2_ polymers with varying molecular weights ranging from 3–17 kDa were prepared, denoted as polymers 3k, 5k, 11k, and 17k. ^1^H NMR and FTIR were used for characterizing the poly­(oxazoline)­s as well as checking the efficiency of Boc-group deprotection. As shown in Figure S3, methyl groups in the Boc that appear at a 1.4 ppm chemical shift all disappeared, indicative of complete Boc deprotection to yield free amines. The ^1^H NMR peaks of the ene functional groups showed up at 5.8 and 5.1 ppm, which were used for the end-group analysis to determine the chemical formula as well as the molecular weight of the polymers (Figure S3). FTIR has also confirmed complete Boc deprotection (Figure S4). Poly­(oxazoline)­s contain two types of C=O groups before Boc removal: a C=O ester from the Boc, showing up at ∼1700 cm^–1^, as well as a C=O amide on the polymer main chain at ∼1630 cm^–1^. After the reaction, the first vibration at ∼1700 cm^–1^ disappeared, whereas the second peak shifted slightly to ∼1625 cm^–1^ (Figure S4).

To covalently attach the polymers to the NaH-ZNT support, a grafting-to approach was adopted by carrying out thiol–ene click reactions.^43^ After the modification of NaH-ZNT with MPTMS, there are excess –SH groups that can react with ene groups from the polymers. In the presence of a 2,2-dimethoxy-2-phenylacetophenone (DMPA) photoinitiator under 365 nm ultraviolet (UV) light, poly­(oxazoline)­s were successfully attached onto the NaH-ZNT-SH support. N_2_ sorption isotherms indicated a further decrease of the BET surface areas and DFT pore volumes (Figure 1a and Table 1). However, the average pore sizes of the polymer-grafted samples did not appreciably change compared to NaH-ZNT-SH (Table 1), indicating that the pore structures of the support remained intact during polymer grafting and that the mesopores were only modestly filled. In addition, as shown in Figure 1c, NaH-ZNT-SH showed extra mass loss compared with NaH-ZNT due to added –SH functional groups (∼2.3 mmol/g), and additional mass loss was further detected after polymer grafting. Elemental analysis was carried out to measure the S and N content across all the samples, and the N content was used for calculating the polymer loadings. As summarized in Table 2, all four samples have a polymer loading less than 20 wt %, which is likely the reason why the pore size did not change after polymer grafting due to their relatively small mass contributions to the samples. However, the Si/N atomic ratios vary greatly between SEM-EDS and XPS (Table 2). Considering that the detection depth of SEM-EDS is typically a few micrometers compared to a few atomic layers in the case of XPS, it can be hypothesized that the majority of the poly­(oxazoline)­s reside inside the NaH-ZNT-SH mesopores. FTIR has also confirmed the successful incorporation of poly­(oxazoline)­s in the ZNT samples. As shown in Figure 1d, after –SH modification and polymer grafting, a –CH_2_– stretch appeared in the region between 2840 and 2980 cm^–1^, while the lower wavenumber regions ∼1440 cm^–1^ are from –CH_2_– bending. The strong signals at 1040 cm^–1^ are from the Si–O–Si vibrations, with the peaks at ∼540 cm^–1^ indicative of NaH-ZNT pentasil structural units. Coincidentally, the poly­(oxazoline) C=O overlaps with the water vibration after being adsorbed on the zeolite at ∼1630 cm^–1^.^44–46^

**Table 2:** Sample Information of NaH-ZNT, NaH-ZNT-SH, and NaH-ZNT-Polymer Composites

sample	S content^a^ (mmol/g)	N content^a^ (mmol/g)	–NH_2_ content^b^ (mmol/g)	[H^+^] content^c^ (mmol/g)	polymer loading^d^ (wt %)	Si/Al^e^	Si/S^e^	Si/N^e^	Si/N^f^
NaH-ZNT	N/A	N/A	N/A	0.15	N/A	14.4	N/A	N/A	N/A
NaH-ZNT-SH	2.03	N/A	N/A	0.11	N/A	17.5	9.6	N/A	N/A
NaH-ZNT-17k	1.44	0.89	0.17	0.10	10.0	18.3	8.6	18.7	9.2
NaH-ZNT-11k	1.34	1.52	0.12	0.09	19.0	18.6	8.9	15.1	3.0
NaH-ZNT-5k	1.17	1.28	0.19	0.11	12.3	16.3	11.2	20.4	4.2
NaH-ZNT-3k	1.68	0.81	0.19	0.10	8.1	17.6	6.2	18.9	11.1

a Determined through combustion CHNS elemental analysis.

b Calculated based on the N content from CHNS elemental analysis.

c NaH-ZNT [H^+^] was determined by IPA-TPD experiments, other samples were adjusted based on the S and N loadings.

d Calculated based on the N content from CHNS elemental analysis.

e Determined by SEM-EDS, the average number was taken from four different areas at low magnification (2000×).

f Determined by XPS.

### Kinetic Study of the NaH-ZNT-Polymer Composite Catalyzed Reactions

The catalytic performance of the bifunctional NaH-ZNT-polymer composites was evaluated by using the model deacetalization-Knoevenagel condensation (Scheme 2). As discussed earlier, NaH-ZNT-polymer composites consist of NaH-ZNT-SH with covalently bonded amine-functionalized poly­(oxazoline)­s, where molecular weight ranges from 3 to 17 kDa. NaH-ZNT-SH provides Brønsted acidic sites that catalyze the first reaction, the deacetalization of benzaldehyde dimethyl acetal to yield benzaldehyde, which acts as a reactant for the second reaction. Amine-functionalized poly­(oxazoline)­s have basic active sites that catalyze the Knoevenagel condensation between benzaldehyde and benzoylacetonitrile to form chalcone ((*E*)-2-benzoyl-3-phenylacrylonitrile: Scheme 2). The reaction solvents were chosen as CDCl_3_/DMSO-*d*_6_ with a 1:1 volume ratio, which favors the solubilizing of poly­(oxazoline)­s as well as facilitating the Knoevenagel condensation.

**Scheme 2:**
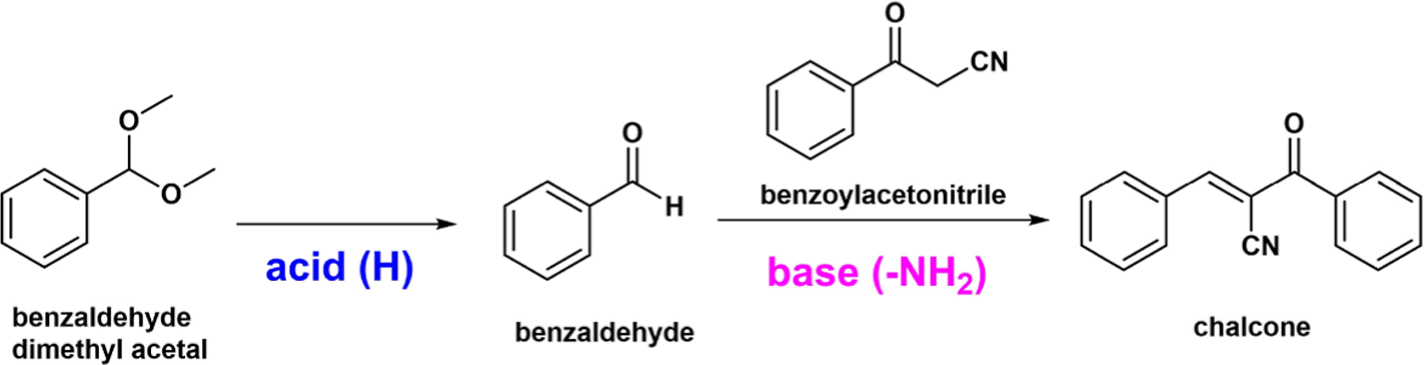
Schematic Illustration of the Model Reaction, Acid-Catalyzed Deacetalization, and Base-Catalyzed Knoevenagel Condensation

The acid-catalyzed and base-catalyzed reactions were first examined separately to study their activities in a single catalytic setup. As shown in Figure S5, both NaH-ZNT and NaH-ZNT-SH showed activity for deacetalization, reaching over 90% conversion of the acetal in 6 h. For the NaH-ZNT sample at 6 h, even though the conversion of the acetal had reached 90%, the yield of benzaldehyde in solution had only reached ∼70% (Figure S5a), possibly due to benzaldehyde being strongly adsorbed on the NaH-ZNT polar surface (–OH). Thus, not all formed benzaldehyde was detected in solution by NMR, a trend that was not observed for the NaH-ZNT-SH sample. In fact, as shown in Figure S6, the experiment using NaH-ZNT probing its basic activity showed that no chalcone was detected, indicative of a lack of basic active sites in the NaH-ZNT materials. Despite this, the benzaldehyde concentration decreased, further supporting the ready adsorption of benzaldehyde on NaH-ZNT surfaces (Figure S6c). The turnover frequency (TOF) of the NaH-ZNT-SH sample in the acid-catalyzed deacetalization was 3.7 × 10^–3^ s^–1^, more than double that of NaH-ZNT, possibly because benzaldehyde can desorb faster from the NaH-ZNT-SH surface than the parent zeolite sample. After the acid activity test, both NaH-ZNT and NaH-ZNT-SH were recovered by centrifugation and washed with copious amounts of dichloromethane before reactivation at 80 °C under a vacuum for use in recycle reactions. N_2_ sorption confirmed the structural integrity of NaH-ZNT and NaH-ZNT-SH after recycling. Shown in Figure S7, the overall shape of the isotherms was unchanged and the surface areas and pore volumes were mostly retained after recycling of NaH-ZNT and NaH-ZNT-SH (Table S2). Compared to NaH-ZNT, whose surface area decreased from 1215 m^2^/g to 920 m^2^/g, the –SH modified analogue seemed to possess higher stability under the testing conditions, where the changes in the surface area and pore volume were negligible. However, even though the particle morphology remained unchanged after recycling (Figure S8), a significant decrease in acid activity was recorded for both samples in the second run (Table S1). As shown in Figure S9, the IR band ∼670 cm^–1^ disappeared in both the NaH-ZNT and NaH-ZNT-SH samples after recycling, which originally comes from the Si–O/Al–O symmetrical stretch.^47,48^ We hypothesize that the disappearance of the Si–O/Al–O symmetrical stretch is indicative of a minor structural degradation occurring during the catalytic testing, leading to the reduced activity upon recycle. Therefore, further recyclability studies of the composites were not pursued. For comparison, we prepared Al-MCM-41 and Al-MCM-41-SH with similar Si/Al (∼15) compared to that of the NaH-ZNT. As shown in Figure S5 and Table S1, their acid catalysis rates were much lower compared to NaH-ZNT and NaH-ZNT-recycled, which indicates that the NaH-ZNT has high activities consistent with zeolite domains for the acid-catalyzed deacetalization.

In parallel, all four –NH_2_ polymers were tested for base-catalyzed Knoevenagel condensation prior to supporting them on the ZNT materials. As summarized in Figure S10 and Table S3, polymer-17k showed the lowest activity in this base-catalyzed reaction, with a TOF of 0.39 × 10^–3^ s^–1^, which is likely caused by its high molecular weight leading to slow solvation of the side chains carrying the active sites, leading to an approximately one hour induction period before the reaction accelerated. On the contrary, poly­(oxazoline)­s with lower molecular weights (3–11 kDa) gave much higher activity, with polymer-5k having the highest TOF of 12.9 × 10^–3^ s^–1^. As shown in the kinetic profiles, the chalcone started to form immediately after starting the reactions, indicating high activity and easy accessibility of the basic –NH_2_ sites in the polymer side chains (Figure S10b–d).

The combined acid–base catalytic tests were carried out in the presence of the various ZNT samples using benzaldehyde dimethyl acetal and benzoylacetonitrile (Scheme 2). The full reaction kinetic profile is shown in Figure 3. All four poly­(oxazoline)-grafted samples achieved ∼100% conversion of benzaldehyde dimethyl acetal in less than 8 h. Compared with NaH-ZNT-SH before polymer grafting, reductions in the initial rates of the acid-catalyzed reaction and TOFs were observed, likely due to the interaction between Brønsted acidic sites and –NH_2_ groups (Table 3). Within the composites, the Brønsted acidic sites are confined on the nanotube pore wall in the micro- and mesopores, and the –NH_2_ groups are on the poly­(oxazoline) side chains. These constraints suggest that the loss of their relatively high catalytic activities relative to cases where they were tested separately (ZNT for acid step, unsupported polymers for base step) is not entirely due to site quenching but also due to reduced accessibility to active sites caused by diffusion or adsorption limitations in the organic/inorganic composite catalysts. As shown in Table 3, the initial acid and base reaction rates of the composites were all approximately the same for the various grafted polymers. In all cases. The TOFs were higher for the grafted polymers than for the corresponding impregnated or mixed polymers (see below). This suggests that chemical tethering limits the ability of the amine polymer to adsorb in a multidentate manner to the nanotube surface and/or block zeolite micropores.

**Figure 3:**
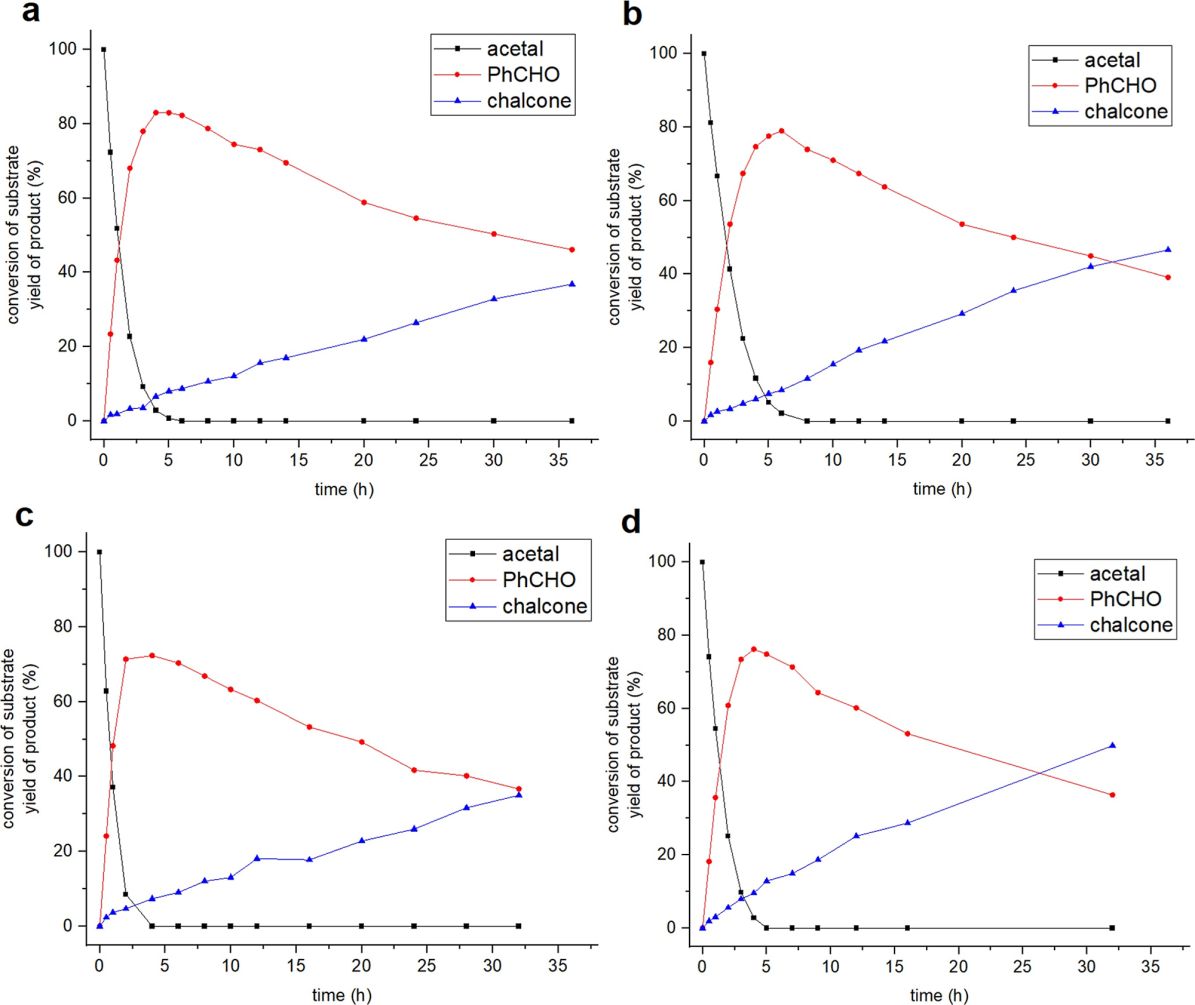
Full kinetic profiles of (a) NaH-ZNT-17k, (b) NaH-ZNT-11k, (c) NaH-ZNT-5k, (d) NaH-ZNT-3k for the acid–base cascade reactions.

**Table 3:** Initial Rates and TOFs of NaH-ZNT-Poly­(oxazoline) Composites and NaH-ZNT-Mix-Poly­(oxazoline)­s for the Combined Acid–Base Cascade Reactions

samples	initial rate acid rxn. (×10^–3^ M h^–1^)	TOF (10^–3^ s^–1^) acid rxn	initial rate base rxn. (×10^–3^ M h–1)	TOF (10^–3^ s^–1^) base rxn
NaH-ZNT-17k	31	1.8	0.52	0.02
NaH-ZNT-11k	21	1.3	0.36	0.04
NaH-ZNT-5k	32	1.8	0.75	0.03
NaH-ZNT-3k	24	1.6	0.45	0.02
NaH-ZNT–SH–mix-17k	5.6	0.33		
NaH-ZNT–SH–mix-11k	0.69	0.04		
NaH-ZNT–SH–mix-5k	0.039	0		
NaH-ZNT–SH–mix-3k	1.1	0.07		

Among all of the composite samples, no induction period was detected for the acid- or base-catalyzed reactions, where benzaldehyde formed during the deacetalization immediately took part in the subsequent Knoevenagel condensation, judging by the steep curve of the benzaldehyde formation and rapid occurrence of chalcone formation (Figure 3). However, the overall, integrated chalcone formation rate was slow, with the highest yield of ∼50% for NaH-ZNT-3k after 30 h. Decreases of the rate of the base-catalyzed reaction using the composite catalysts were confirmed. Compared to the polymers alone, the rates of the base-catalyzed reaction of the composites were dramatically lower. All four samples were also tested for the base-catalyzed half-reaction (Figure S11) alone. Compared to the unsupported polymers (Table S3), sharp decreases in the initial reaction rates were observed for all of the samples, confirming that the interaction between Brønsted acidic sites and –NH_2_ groups has led to the decreased activity of the polymers for the base-catalyzed reaction.

To further understand the interaction(s) between NaH-ZNT-SH and poly­(oxazoline)­s in the grafted materials, physical mixtures of NaH-ZNT-SH and poly­(oxazoline)­s were prepared as reference samples and tested under the same conditions. As shown in Figure S12 and Table 3, the acid catalytic activities of the physical mixtures of NaH-ZNT-SH and poly­(oxazoline)­s were decreased further compared with the catalysts prepared by covalently grafting the polymers on the catalysts (Figure 3 and Table 3). In addition, an induction period of approximately 1 h was observed using the physical mixture of NaH-ZNT-SH and the 17k, 11k, 3k and polymers, respectively. For the sample NaH-ZNT-SH mixed with polymer-5k, the base catalytic activity was reduced to zero, indicating that all of the –NH_2_ groups have been deactivated by Brønsted acidic sites (Figure S12c). The initial acid reaction rates of physical mixtures of NaH-ZNT-SH and the poly­(oxazoline) polymers were lower than the grafted composites in all cases. Considering the reactions were performed at 60 °C under continuous stirring, it is possible that for the physical mixtures, polymers slowly move on/off the zeolite surface or diffuse out from the nanotube mesopores during the reaction. As noted above, the physical mixture of NaH-ZNT-SH and polymer-5k has near zero activity in the combined acid–base cascade reactions (Table 3). We hypothesize that in the case of the NaH-ZNT-mix-5k sample, the size of the polymer is very close to the mesopore size of the nanotube, and therefore, it interacted with the pore wall most efficiently and almost quenched the activity of the nanotube acid sites. As shown Table S4, polymer-5k has a high initial rate when tested for the base-catalyzed half reaction, indicative of a high amine activity. Therefore, when not surface-grafted, as for NaH-ZNT–SH–mix-5k, its low activity suggests significant surface adsorption, leading to acid/base quenching reactions and steric constraints that led to near zero activity. The acid contents of NaH-ZNT, NaH-ZNT-5k, and NaH-ZNT–SH–mix-5k were also measured by acid titration experiments. As shown in Table S5, NaH-ZNT-mix-5k has a much lower acid content compared to NaH-ZNT-5k. Since NaH-ZNT-5k had the highest initial reaction rates among the grafted composites, the grafting-to approach for preparing the zeolite nanotube composites is demonstrated to provide higher efficacy for cascade catalysis, especially when the strong interaction between competing active sites cannot be avoided. As summarized in Table S6, all of the composites except NaH-ZNT–SH–mix-5k show moderate activity, yielding more than 30% chalcone compounds in 30 h, with the grafted composites providing additional advantages including ease of separation and faster initial reaction rates. However, none of the materials offer state-of-the-art performance for the target reaction cascades, which can reach completion within hours.^8^

## Conclusions

In this study, we synthesized NaH-ZNT-poly­(oxazoline) composite materials and evaluated their potential for liquid-phase acid–base cascade catalysis for the synthesis of chalcone. The NaH-ZNT provides Brønsted acidic sites that are active for acid-catalyzed reactions and also acts as supports for polymer grafting, providing additional catalytic functionalities. Poly­(oxazoline)­s with different molecular weights were prepared and attached to NaH-ZNT via a grafting-to approach. The results suggest that the interactions between NaH-ZNT and the poly­(oxazoline)­s vary depending on the polymer molecular weights, where the polymer of 5 kDa interacts most strongly with the NaH-ZNT, deactivating all the active sites. On the contrary, the corresponding grafted composite exhibited the highest activity, though some site-quenching interactions between NaH-ZNT and amine polymer cannot be avoided. Overall, this work demonstrates the feasibility of using NaH-ZNT-poly­(oxazoline) composites for cascade catalysis for producing organic compounds and highlights the importance of fine-tuning the interactions between competing catalytic sites to maximize the efficiency.

## Supplementary Material



## Notes

Reaction kinetic profiles, reaction rates, IR spectra, 1H NMR spectra, N2 physisorption data, and SEM images (PDF)
