# Supplementary material for: Single-Walled Zeolitic Nanotube–Poly(oxazoline) Nanocomposites as Heterogeneous Catalysts for Acid–Base Cascade Reactions
Source: Langmuir. 2025 May 16;41(20):12772–80. doi: 10.1021/acs.langmuir.5c01067 (PMC12120977; doi:10.1021/acs.langmuir.5c01067)
Supplement: Supplementary file 1 [file la5c01067_si_001.pdf]

*Supporting Information for:*

# Single-Walled Zeolitic Nanotube–Poly(oxazoline) Nanocomposites as Heterogeneous Catalysts for Acid-Base Cascade Reactions

Wenyang Zhao, Anthony Vallace, Younhwa Kim, Christopher W. Jones*

*School of Chemical & Biomolecular Engineering, Georgia Institute of Technology, 311 Ferst Drive, Atlanta, GA 30332, United States*

*cjones@chbe.gatech.edu

## Table of Contents

1. **Materials**
2. **Instruments**
3. **Methods**
4. **Additional Data**

## 1. Materials

2-(4-((*tert*-Butoxycarbonyl)amino)butyl)-2-oxazoline,[1] *R*-2-butyl-4-ethyl-2-oxazoline,[2] and proton-exchanged zeolite nanotube (NaH-ZNT)[3] were synthesized based on adapted literature procedures. All reagents were purchased from standard suppliers and used as received unless otherwise stated. 2-Methyl-2-oxazoline, acetonitrile, chlorobenzene were distilled over $CaH_2$ and stored under dry Ar and molecular sieve (4 Å). Methyl triflate (MeOTf) was distilled over BaO and stored under dry Ar at -20 °C.

## 2. Instruments

$N_2$ sorption experiments were conducted using a Micromeritics Tristar $N_2$ analyzer at 77 K. Prior to analysis, samples were activated at ~12 mTorr at 80 °C for 10 h. BET surface areas were calculated using the data points between 0.05–0.35 $P/P_0$. Pore volumes and average pore sizes were calculated by applying NLDFT with a siliceous model.

Thermogravimetric analysis (TGA) was performed using a TGA 550 Discovery Series from TA Instruments. Data were recorded with a ramp rate of 10 °C/min under $N_2$ atmosphere from room temperature to 100 °C, after a 1 h isothermal step at 100 °C, then started heating again in air with the same ramp rate of 10 °C/min to 700 °C. The organic content was estimated from the loss in the 180–650 °C temperature range.

Fourier-transform infrared spectroscopy (FTIR) was performed with a Nicolet 6700 FT-IR spectrometer equipped with a diamond crystal Attenuated Total Reflectance (ATR) attachment. Spectra were recorded from 4000–500 $cm^{-1}$ with a total number of 256 scans. Backgrounds were collected before each spectrum acquisition.

Elemental analyses (EA) were carried out by combustion method for all the samples, and analyzed for carbon, hydrogen, nitrogen, and sulfur contents. Analyses were performed by Atlantic Microlab at Norcross, GA, 30071.

Scanning transmission electron microscopy (STEM) was performed using a Hitachi HD2700 with an 80 kV accelerating voltage and a spherical aberration (Cs) corrected cold field emission source.

Samples were prepared by drop-casting ethanol suspension onto carbon film coated lacey carbon supported copper grid.

A Hitachi SU8230 scanning electron microscope (SEM) was used for sample imaging and energy dispersive spectroscopy (EDS). An accelerating voltage of 5 kV was used for normal imaging, and 20 kV was used for EDS acquisition.

Nuclear magnetic resonance ($^1$H NMR) spectra were collected via Bruker AV3 HD 700 MHz and Bruker AV3 400 MHz NMR spectrometer. For polymer samples, the D1 parameter was increased to 10 seconds and 128 scans were recorded for proper end-group analysis.

## 3. Methods

### NaH-ZNT synthesis

Zeolite nanotube (NaH-ZNT) synthesis, using NaOH as a mineralizer, was performed using our recently published procedure.[3] First, 0.113 g of BCPH10Qui and 4.45 g of deionized water was added to a 30 mL polypropylene bottle and was stirred to obtain a homogeneous suspension. Next, 0.067 g of NaOH and 0.027 g of aluminum sulfate hydrate ($Al_2(SO_4)_3 \cdot 14–18H_2O$) was dissolved in the reaction mixture. Lastly, 0.5 g of Ludox SM-30 colloidal $SiO_2$ was added, giving a final gel composition of $18.75SiO_2$: 1BCPH10Qui: $0.3Al_2O_3$: $6.3Na_2O$: $2050H_2O$. The gel was aged at room temperature with stirring for 3 h and then was transferred to a Teflon-lined autoclave and allowed to crystallize for 7 days at 150 °C. The resulting solid separated with centrifugation, washed three times with deionized water, and dried in an oven at 75 °C overnight. The solid was then calcined in stagnant air at 550 °C (2 °C/min) for 6 h.

### Polymer synthesis

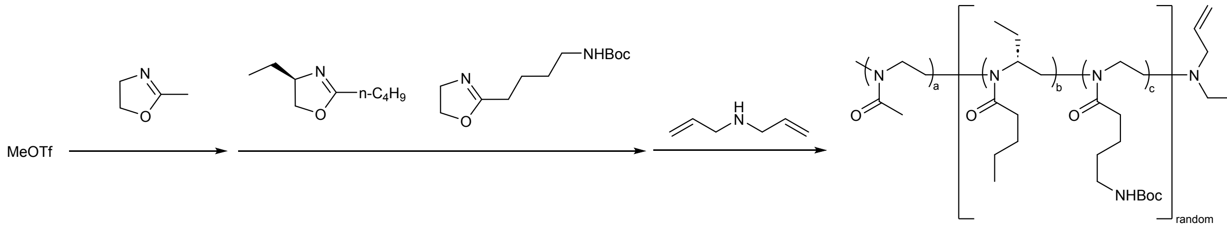

The synthesis of poly(oxazoline) triblock copolymers was adapted from a previous reported procedure.[4] In a typical synthetic procedure, methyl triflate (MeOTf, 54.7 μL, 0.5 mmol) was added to a solution of 2-methyl-2-oxazoline (1.06 mL, 12.5 mmol) in chlorobenzene (6 mL) and acetonitrile (4 mL). The mixture was stirred for 12 h at 80 °C, the reaction progress was monitored by NMR based on the disappearance of the peaks at 3.8 and 4.2 ppm. After 2-methyl-2-oxazoline was fully consumed, the reaction temperature was increased to 110 °C. A mixture of 2-(4-((tert-butoxycarbonyl)amino)butyl)-2-oxazoline (606 mg, 2.5 mmol), *R*-2-butyl-4-ethyl-2-oxazoline (776 mg, 5.0 mmol) and chlorobenzene (10 mL) was added. The solution was stirred at 110 °C for another 3 days. The temperature was then decreased to 90 °C, followed by the addition of diallylamine (124 μL, 1.0 mmol), and reacted for another 24 h. Due to use of the bulky *R*-2-butyl-4-ethyl-2-oxazoline, which slowed down the polymerization reaction during propagation, typically only ~60–70% of that monomer was converted and incorporated into the polymer. The polymer was purified by dialysis against EtOH:$H_2O$ = 2:1 (v/v), and dried under 12 mTorr vacuum at room temperature for 12 h.

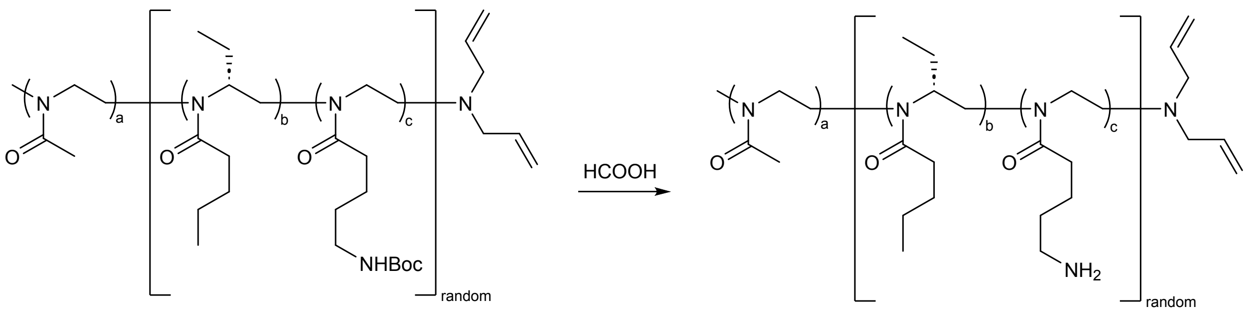


For the Boc deprotection (above), ~200 mg of polymer obtained from the dialysis was dissolved in 4 mL dry dichloromethane (DCM). Then, 2 mL formic acid (HCOOH) was added, and the mixture was stirred vigorously at room temperature for 3 days. Upon completion, the majority of HCOOH was removed using rotary evaporator together with DCM. The residual was dissolved in 4 mL DCM followed by the addition of 2 mL $NH_3 \cdot H_2O$. The mixture was again stirred vigorously at room temperature for 3 days. Additional DCM (~30 mL) was added later to extract the polymer to the organic layer. The polymer in DCM solution was then collected and dried under 12 mTorr vacuum at room temperature for 12 h. The repeating units and chemical formula were determined by NMR spectroscopy via end-group analysis.

## NaH-ZNT-SH synthesis

First, 160 mg NaH-ZNT was dried under 12 mTorr vacuum at 120 °C for 12 h. After cooling to room temperature, 20 mL dry toluene was added to disperse the powder in the suspension. Next, 68 μL (3-mercaptopropyl)trimethoxysilane (MPTMS) was added, and the mixture was refluxed at 100 °C for 12 h. After cooling to room temperature, the solid powder was collected by centrifugation. Subsequently, 30 mL dry acetone was added to disperse the powder and wash it to remove unreacted MPTMS. The suspension was centrifuged again to obtain the product powder. This process was repeated 3 times. After final centrifugation, the powder was collected and dried under 12 mTorr vacuum at 80 °C for 12 h.

## NaH-ZNT-polyoxazoline synthesis

The thiol–ene click reaction procedure was adapted from another literature report.[5] First, 50 mg NaH-ZNT-SH was mixed with 10 mol% (relative to the SH content) 2,2-dimethoxy-2-phenylacetophenone (DMPA) photoinitiator in a 20-mL vial. Next, the appropriate amount of poly(oxazoline) was dissolved in 8 mL dry DCM, and added into the vial. The amount of polymer added was determined based on the thiol loading, with the goal of achieving a comparable $-NH_2$:[$H^+$] molar ratio in the final composites. The vial was septum-sealed and purged with ultra-high purity Ar for ~30 min, then exposed to 365 nm UV light for 24 h while stirring at room temperature. The solid was collected via centrifugation, and 20 mL dry DCM was added to wash and disperse the solid, followed by centrifugation. This process was repeated three times. After the final centrifugation, the solid was obtained and dried under 12 mTorr vacuum at 80 °C for 12 h.

## NaH-ZNT-SH polyoxazoline physical mixture preparation

To prepare a physical mixture of zeolite nanotube and poly(oxazoline), a similar procedure was used compared to the thiol–ene click reaction. The same amount of NaH-ZNT-SH, poly(oxazoline)

and DCM were used, where the mixture was stirred at room temperature for 24 h before drying under 12 mTorr vacuum at 80 °C for 12 h.

**Al-MCM-41 synthesis**

The synthesis procedure for the Al-MCM-41 was adapted from a previous report.[6] In a 500-mL round bottom flask, 240 mL water, 1.75 mL 2M NaOH solution, and 500 mg cetyltrimethylammonium bromide were added. The mixture was stirred at 80 °C until a clear solution was formed. 2.5 mL TEOS were later added dropwise. The mixture was stirred at 80 °C for another 2 h followed by vacuum filtration. The solid obtained was dried at room temperature followed by calcination at 550 °C for 6 h to yield MCM-41. Next, 200 mg MCM-41 was dispersed in 20 mL isopropanol by sonication, followed by the addition of 45.3 mg aluminum isopropoxide. The mixture was refluxed at 80 °C for 16 h. After the reaction, the obtained powder was collected by centrifugation and washed with 30 mL dry hexane 3 times. The powder was then dried at room temperature, followed by calcination at 550 °C for 6 h to yield Al-MCM-41.

**Procedure for acid-catalyzed reaction**

In a one-dram vial equipped with a small stir bar, 16.6 mg NaH-ZNT or 19.9 mg NaH-ZNT-SH powder was added. Then, 0.25 mL dry $CDCl_3$, 0.25 mL dry DMSO-$d_6$, 2.5 µL $H_2O$, 2.5 µL 1,3-dimethylbenzene (DMB), 5 µL benzaldehyde dimethyl acetal were added. The vial was placed onto a preheated heating block at 60 °C to begin the reaction. During the reaction, a 2 µL aliquot was collected at each time point to monitor the reaction progress.

**Procedure for base-catalyzed reaction**

In a one-dram vial equipped with a small stir bar, a certain amount of polyoxazoline in DCM solution was added. The amount of polymer used was determined by the $-NH_2$ content which was ~10 mol% of the benzaldehyde. 0.25 mL dry $CDCl_3$, 0.25 mL dry DMSO-$d_6$, 2.5 µL DMB, 3.4 µL benzaldehyde, 5.8 mg benzoylacetonitrile were added. The vial was placed onto a preheated heating block at 60 °C to begin the reaction. 2 µL aliquot was collected each time to monitor the reaction progress.

## Procedure for the combined acid-base cascade reaction

In a one-dram vial equipped with a small stir bar, ~20 mg NaH-ZNT-polyoxazoline powder was added. 0.25 mL dry $CDCl_3$, 0.25 mL dry DMSO-$d_6$, 2.5 μL $H_2O$, 2.5 μL 1,3-dimethylbenzene (DMB), 5 μL benzaldehyde dimethyl acetal, and 5.8 mg benzoylacetonitrile were added. The vial was placed onto a preheated heating block at 60 °C to begin the reaction. 2 μL aliquot was collected each time to monitor the reaction progress.

## Acid content titration experiments

Acid content of NaH-ZNT, NaH-ZNT-5k, and NaH-ZNT-SH-mix-5k are determined by back-titration approach via the following procedure. First, 20 mg of the materials are added to the 16 mL of saturated NaCl solution and stirred at room temperature for 3 h. The solid and the supernatant are collected separately after centrifugation. Afterwards, 2–3 drops of phenolphthalein indicator is added to the supernatant, and the liquid is titrated slowly with 5 mM NaOH solution until the color changes to pink. The acid content is calculated based on the sample mass, volume of NaCl solution, and the volume of 5 mM NaOH solution.

## 4. Additional Data

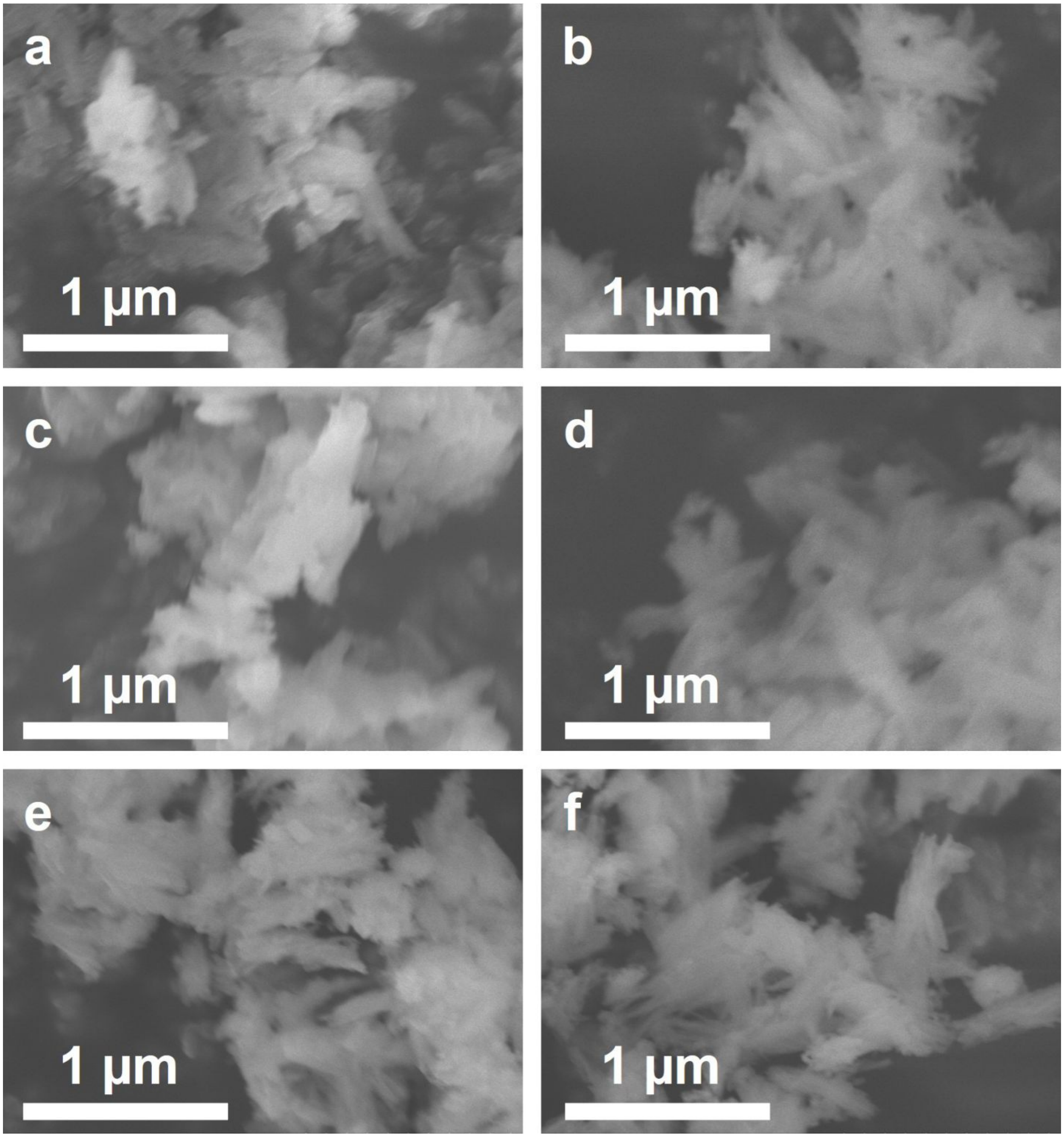


**Figure S1.** SEM images of (a) NaH-ZNT, (b) NaH-ZNT-SH, (c) NaH-ZNT-17k, (d) NaH-ZNT-11k, (e) NaH-ZNT-5k, (f) NaH-ZNT-3k.

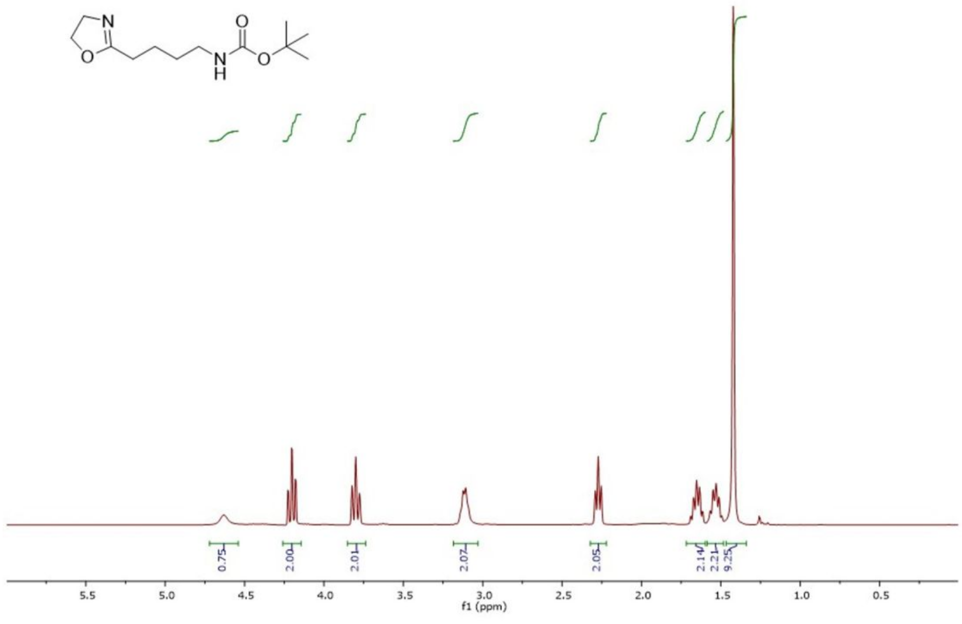

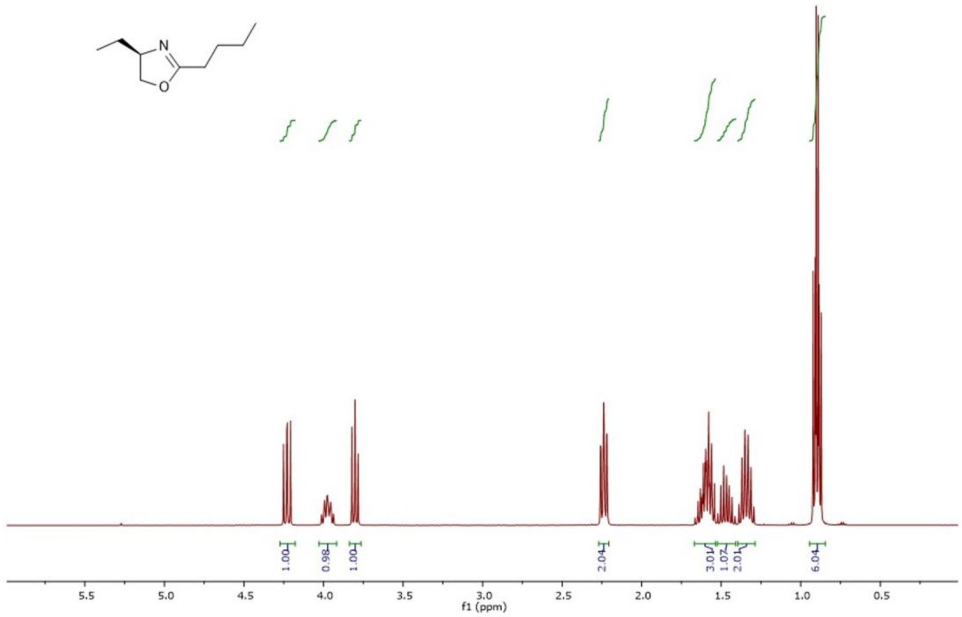

**Figure S2.** $^{1}H$ NMR spectra of two prepared oxazoline monomers.

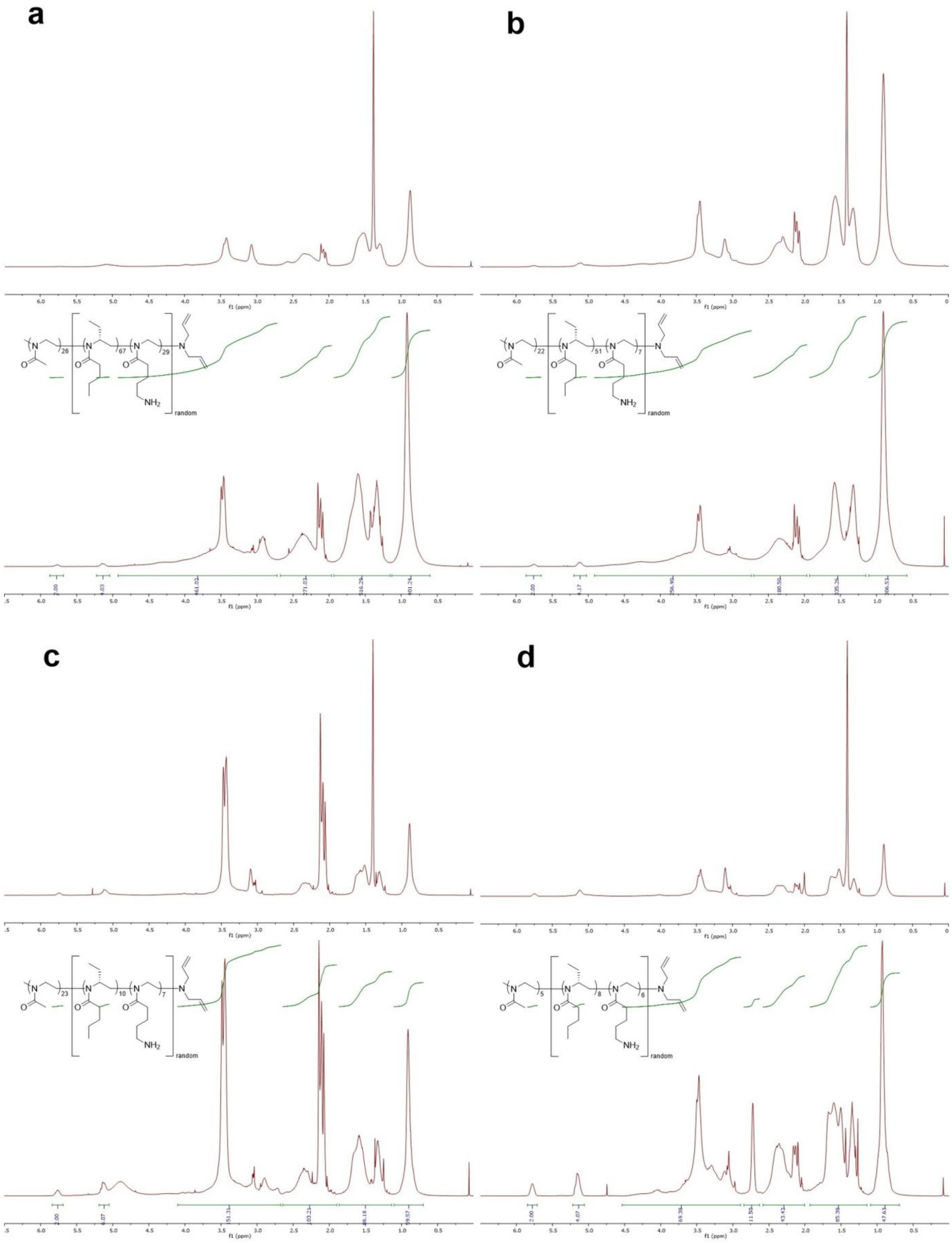

**Figure S3.** $^{1}$H NMR spectra of different poly(oxazoline)s before and after Boc deprotection.

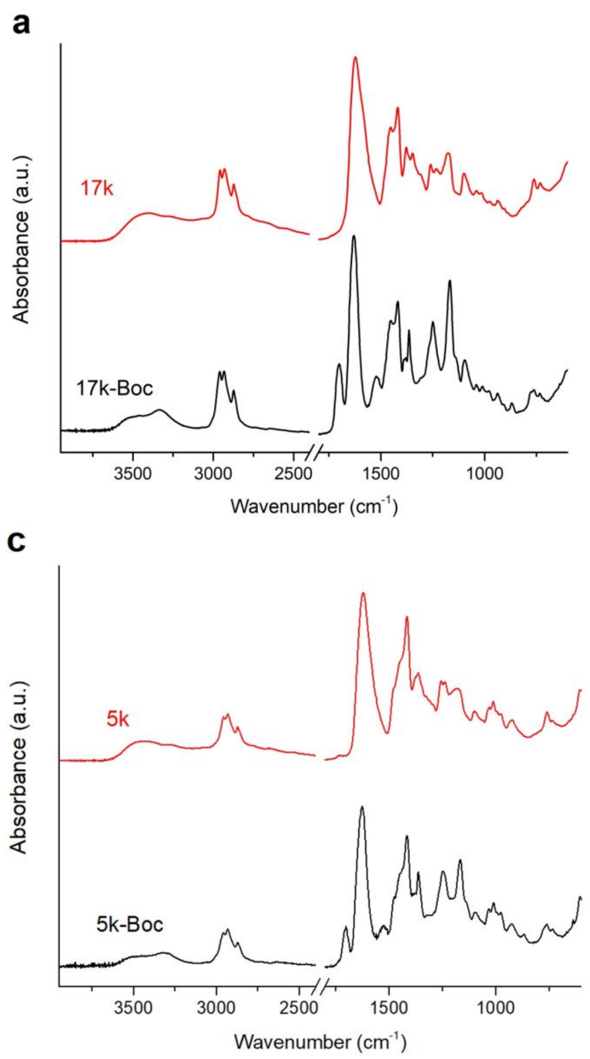


**Figure S4.** FTIR spectra of poly(oxazoline)s before and after Boc deprotection.

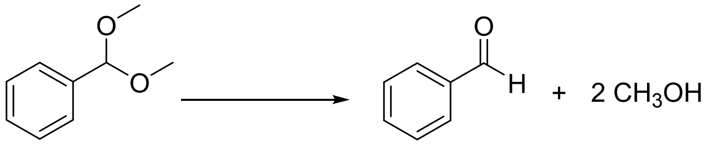


**Scheme S1.** Acid-catalyzed deacetalization reaction.

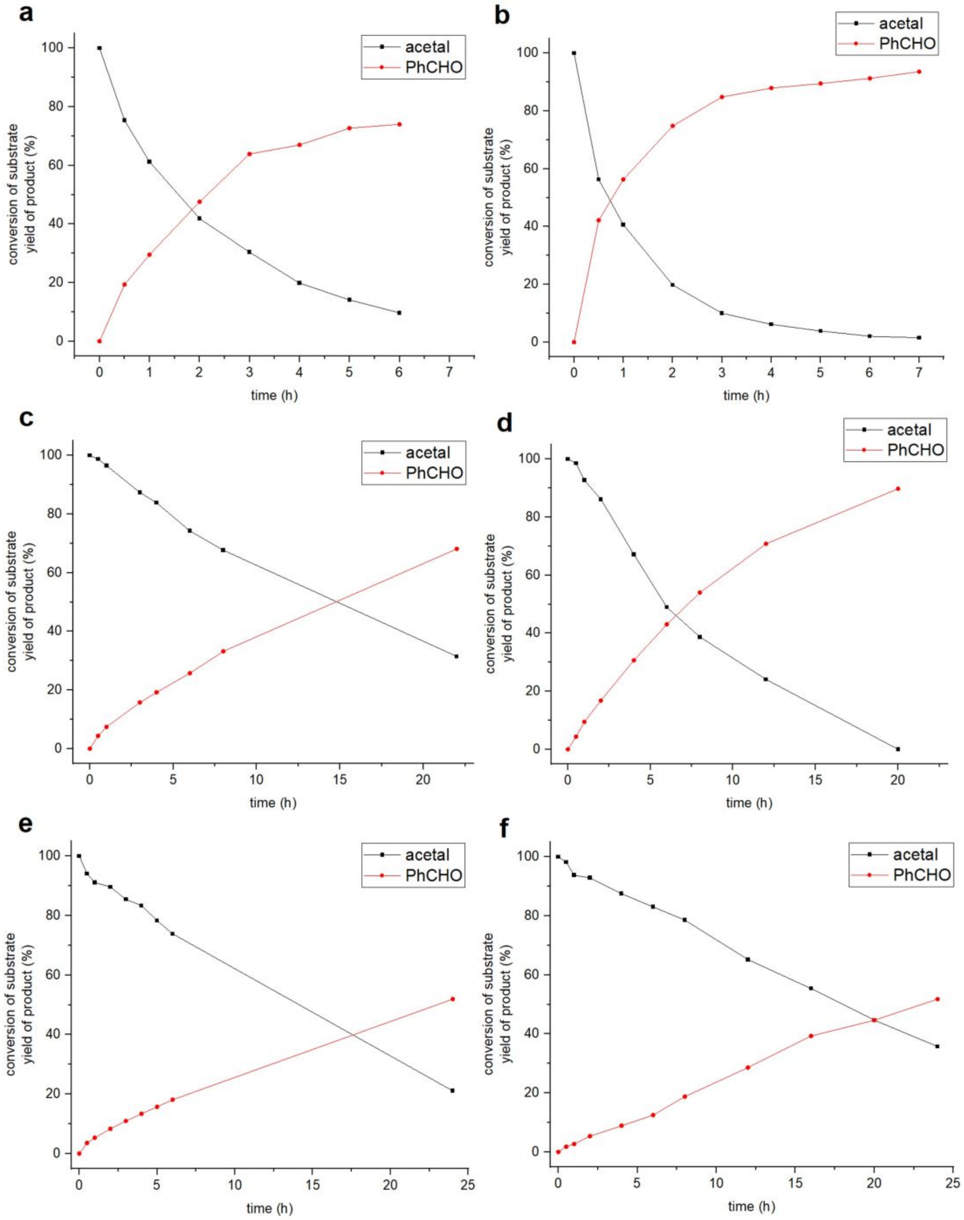


**Figure S5.** Acid-catalyzed reaction profile of (a) NaH-ZNT, (b) NaH-ZNT-SH, (c) NaH-ZNT-recycled, (d) NaH-ZNT-SH-recycled, (e) Al-MCM-41, and (f) Al-MCM-41-SH.

**Table S1.** Summary of the reaction kinetics of the control samples for acid-catalyzed deacetalization.

| Samples for acid rxn. | Initial rate (M $h^{-1}$) | TOF ($10^{-3}$ $s^{-1}$) |
|---|---|---|
| NaH-ZNT | 0.05 | 1.4 |
| NaH-ZNT-SH | 0.11 | 3.7 |
| NaH-ZNT-recycled | 0.0058 | 0.33 |
| NaH-ZNT-SH-recycled | 0.0058 | 0.33 |
| Al-MCM-41 | 0.0011 | N/A |
| Al-MCM-41-SH | 0.00057 | N/A |

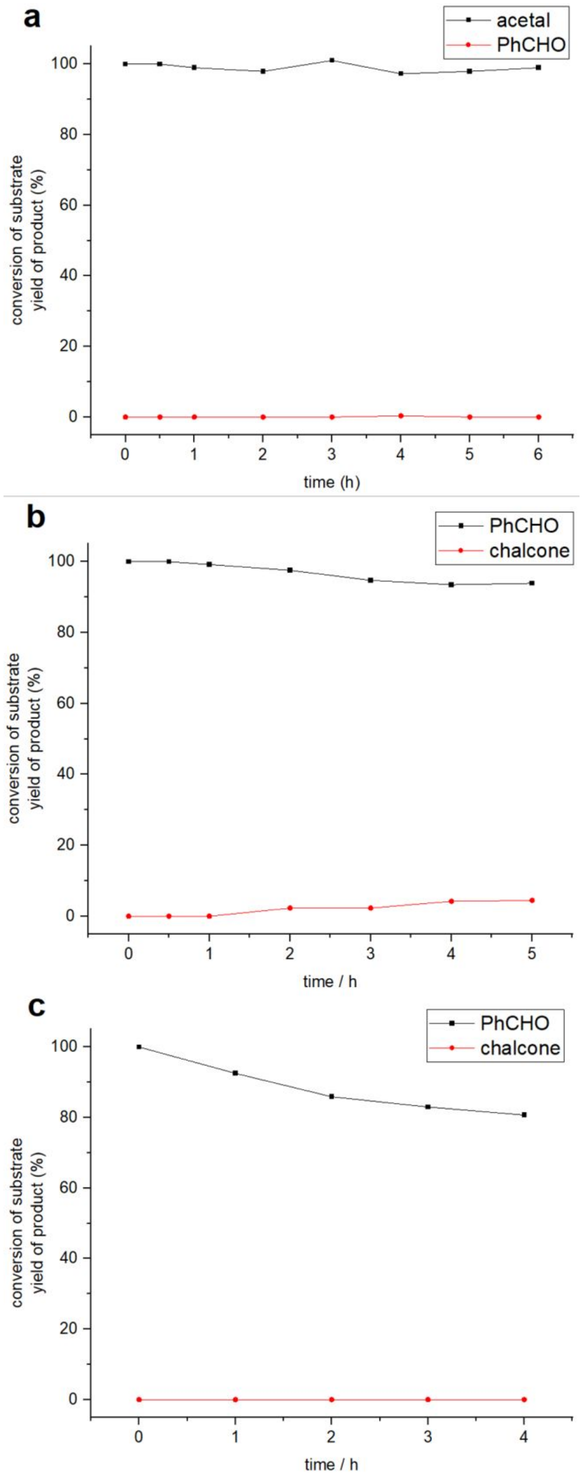


**Figure S6.** Blank catalytic tests of (a) benzaldehyde dimethyl acetal deacetalization without any catalysts, (b) benzaldehyde Knoevenagel condensation without any catalysts, and (c) benzaldehyde Knoevenagel condensation in the presence of NaH-ZNT.

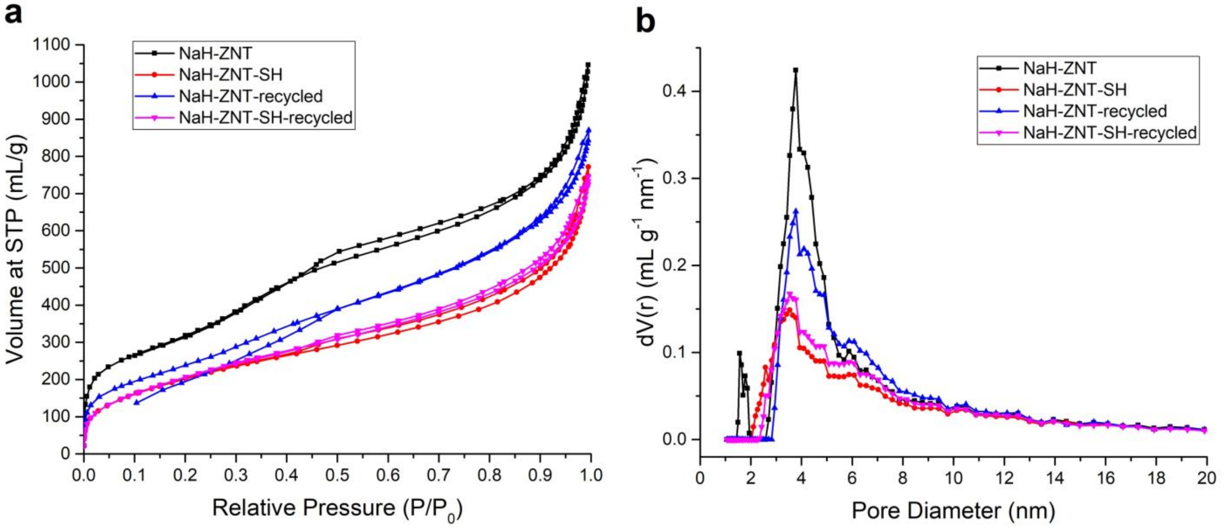


**Figure S7.** (a) $N_2$ sorption isotherms, (b) DFT pore size distributions. Pore size distributions were calculated by NLDFT using a siliceous model.

**Table S2.** Summary of the textual properties of the control samples.

| Sample | BET Surface Area ($m^2$/gNa-ZNT) | DFT Pore Volume (mL/gNa-ZNT) | DFT Average Pore Size (nm) |
|---|---|---|---|
| NaH-ZNT | 1215 | 1.41 | 3.8 |
| NaH-ZNT-SH | 1025 | 1.35 | 3.5 |
| NaH-ZNT-recycled | 920 | 1.18 | 3.8 |
| NaH-ZNT-SH-recycled | 1085 | 1.37 | 3.5 |

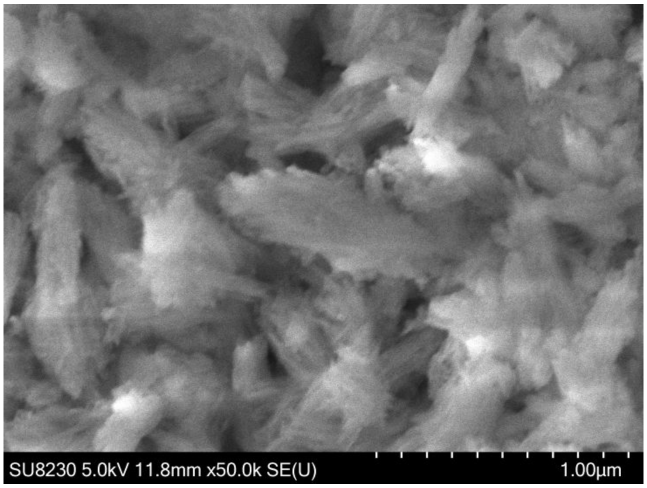


**Figure S8.** SEM image of NaH-ZNT-recycled.

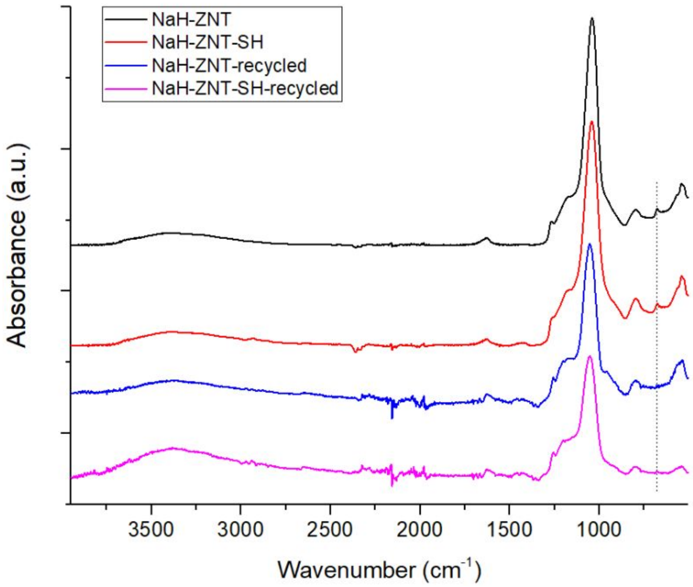


**Figure S9.** FTIR spectra of NaH-ZNT, NaH-ZNT-SH, NaH-ZNT-recycled, and NaH-ZNT-SH-recycled.

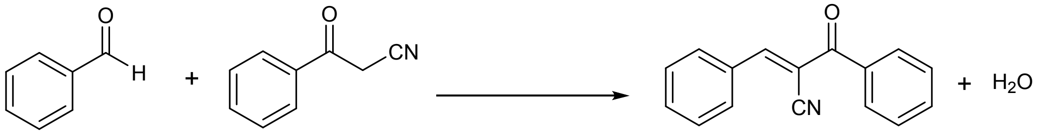


**Scheme S2.** Base-catalyzed Knoevenagel condensation.

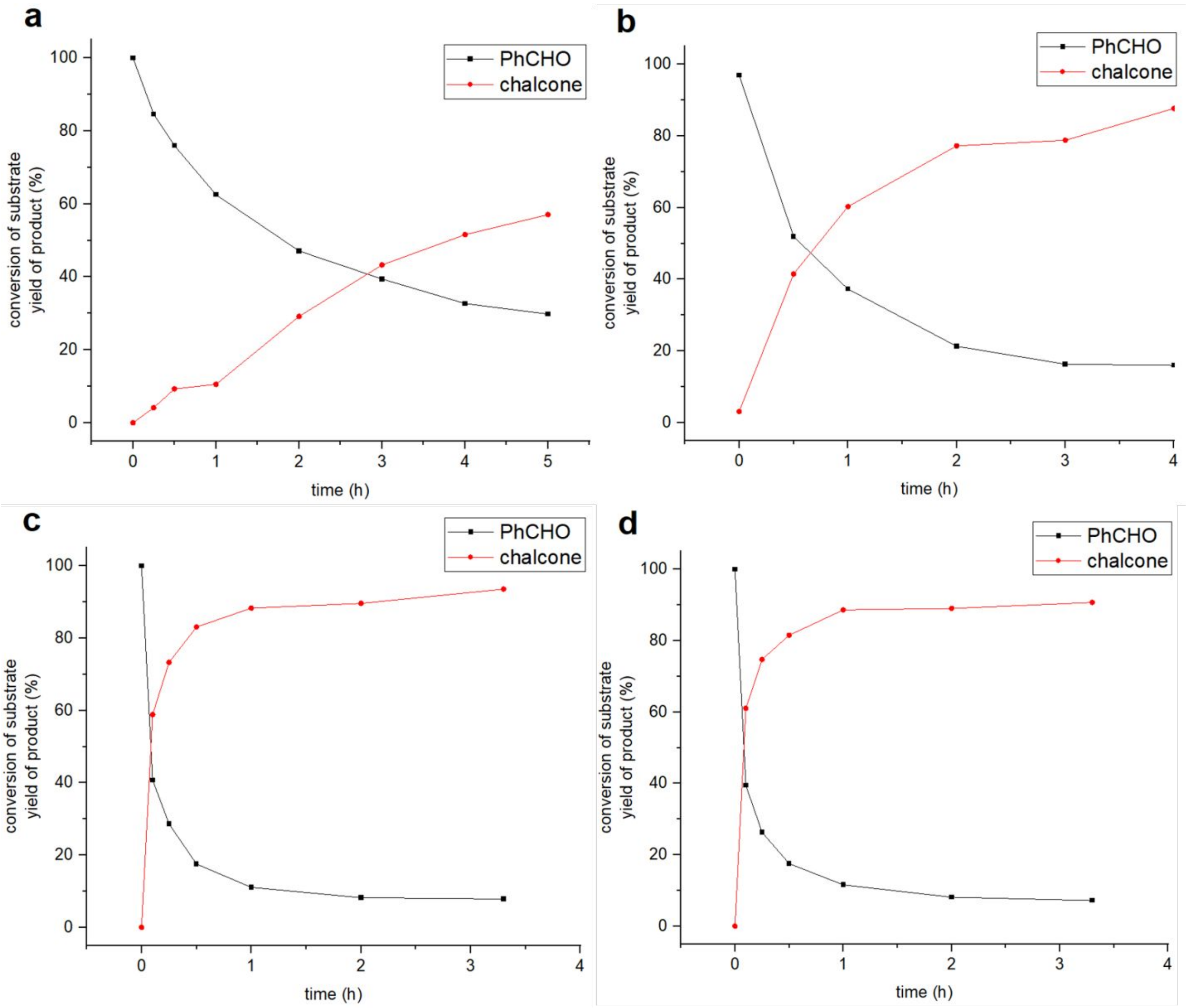


**Figure S10.** Base-catalyzed reaction profile of (a) 17k, (b) 11k, (c) 5k, and (d) 3k unsupported polymers.

**Table S3.** Summary of the reaction kinetics of the control samples for base-catalyzed Knoevenagel condensation.

| Samples for base rxn. | $-NH_2$ content (mmol/g) | Initial rate ($M\ h^{-1}$) | TOF ($10^{-3}\ s^{-1}$) |
|---|---|---|---|
| 17k | 1.7 | 0.01 | 0.4 |
| 11k | 0.65 | 0.055 | 6.7 |
| 5k | 1.5 | 0.39 | 12.9 |
| 3k | 2.3 | 0.41 | 8.3 |

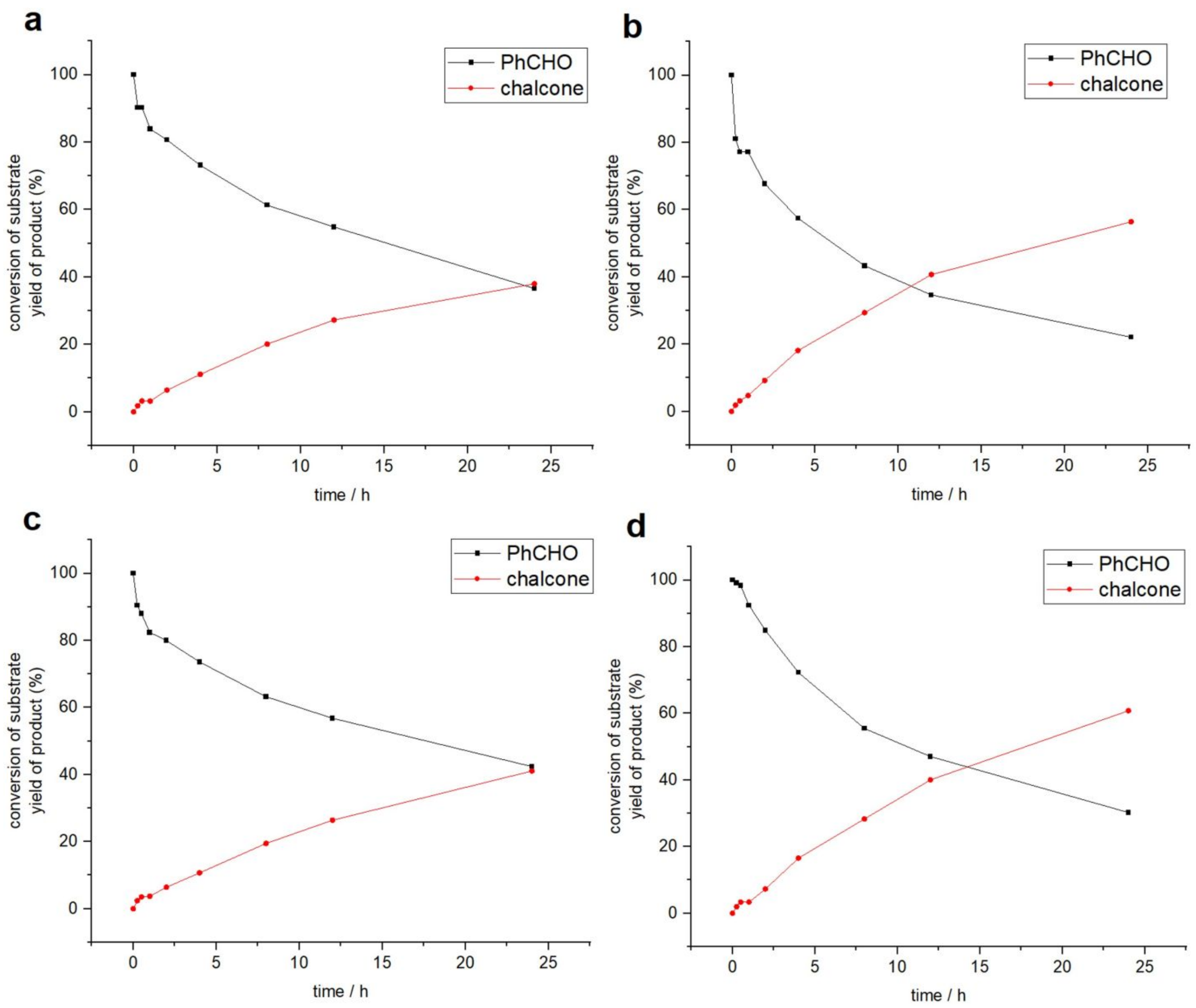


**Figure S11.** Base-catalyzed half reaction profile of (a) NaH-ZNT-17k, (b) NaH-ZNT-11k, (c) NaH-ZNT-5k, and (d) NaH-ZNT-3k.

**Table S4.** Initial rates and TOFs of NaH-ZNT-poly(oxazoline) composites for base-catalyzed half reaction.

| Samples | Initial rate ($\times 10^{-3}$ M $h^{-1}$) | TOF ($10^{-3}$ $s^{-1}$) |
|---|---|---|
| NaH-ZNT-17k | 4.8 | 0.17 |
| NaH-ZNT-11k | 4.9 | 0.59 |
| NaH-ZNT-5k | 6.0 | 0.24 |
| NaH-ZNT-3k | 5.2 | 0.21 |

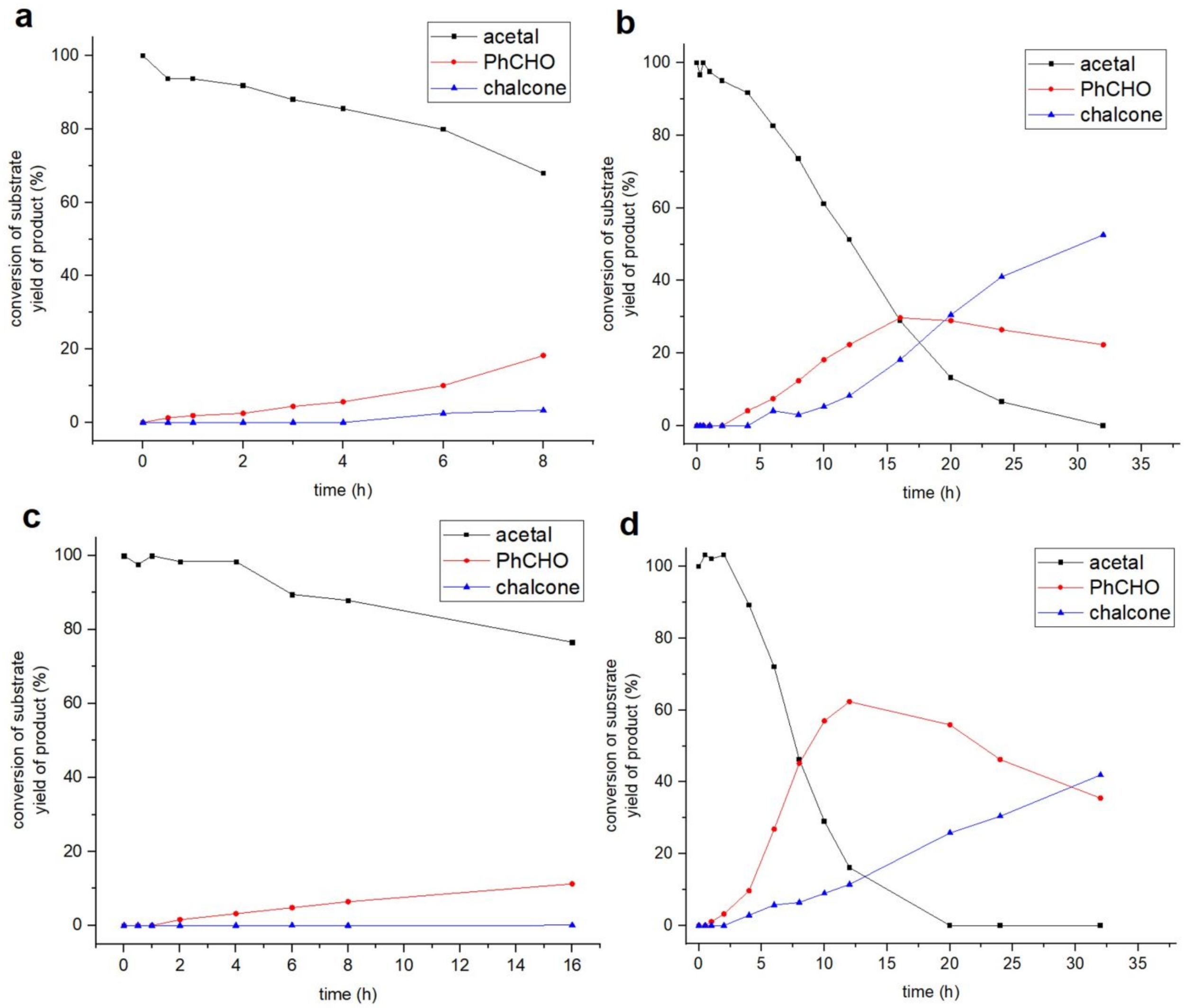


**Figure S12.** Full kinetic profiles of (a) NaH-ZNT-mix-17k, (b) NaH-ZNT-mix-11k, (c) NaH-ZNT-mix-5k, (d) NaH-ZNT-mix-3k for the acid-base cascade reactions.

**Table S5.** Summary of the acid content calculated from titration experiments.

| Samples | Acid content ($mmol/g_{NaH-ZNT}$) |
|---|---|
| NaH-ZNT | 0.46 |
| NaH-ZNT-5k | 0.37 |
| NaH-ZNT-SH-mix-5k | 0.14 |

**Table S6.** The yield of the cascade product chalcone at 30 h.

| Samples | Yield% |
|---|---|
| NaH-ZNT-17k | 33 |
| NaH-ZNT-11k | 42 |
| NaH-ZNT-5k | 33 |
| NaH-ZNT-3k | 47 |
| NaH-ZNT-SH-mix-17k | 33 |
| NaH-ZNT-SH-mix-11k | 50 |
| NaH-ZNT-SH-mix-5k | 0 |
| NaH-ZNT-SH-mix-3k | 47 |

## References


1. Hartlieb, M.; Pretzel, D.; Kempe, K.; Fritzsche, C.; Paulus, R. M.; Gottschaldt, M.; Schubert, U. S., Cationic poly(2-oxazoline) hydrogels for reversible DNA binding. *Soft Matter* **2013,** *9*, 4693.
2. Bloksma, M. M.; Rogers, S.; Schubert, U. S.; Hoogenboom, R., Secondary structure formation of main-chain chiral poly(2-oxazoline)s in solution. *Soft Matter* **2010,** *6*, 994.
3. Korde, A.; Min, B.; Kapaca, E.; Knio, O.; Nezam, I.; Wang, Z.; Leisen, J.; Yin, X.; Zhang, X.; Sholl, D. S.; Zou, X.; Willhammar, T.; Jones, C. W.; Nair, S., Single-walled zeolitic nanotubes. *Science* **2022,** *375*, 62-66.
4. Lee, L.-C.; Lu, J.; Weck, M.; Jones, C. W., Acid–base bifunctional shell cross-linked micelle nanoreactor for one-pot tandem reaction. *ACS Catal.* **2016,** *6*, 784-787.
5. Mai, T. B.; Tran, T. N.; Rafiqul Islam, M.; Park, J. M.; Lim, K. T., Covalent functionalization of silica nanoparticles with poly(N-isopropylacrylamide) employing thiol-ene chemistry and activator regenerated by electron transfer ATRP protocol. *J. Mater. Sci.* **2013,** *49*, 1519-1526.
6. Mokaya, R., Post-synthesis grafting of Al onto MCM-41. *Chem. Commun.* **1997**, *2185-2186.*
